# Effects of creatine supplementation associated with resistance training on oxidative stress in different tissues of rats

**DOI:** 10.1186/1550-2783-11-11

**Published:** 2014-03-24

**Authors:** Giuseppe Potrick Stefani, Ramiro Barcos Nunes, André Zuanazzi Dornelles, Jadson Pereira Alves, Marcella Ody Piva, Marlise Di Domenico, Cláudia Ramos Rhoden, Pedro Dal Lago

**Affiliations:** 1 Laboratório de Fisiologia – UFCSPA/Porto Alegre, Rua Sarmento Leite, 245, 900050-170 Porto Alegre, RS, Brazil; 2 Laboratório de Poluição Atmosférica e Estresse Oxidativo – UFCSPA/Porto Alegre, Porto Alegre, RS, Brazil; 3 Programa de Pós Graduação em Ciências da Reabilitação – UFCSPA/Porto Alegre, Porto Alegre, RS, Brazil

**Keywords:** Creatine, Supplementation, Resistance training, Oxidative stress, Lipoperoxidation, Antioxidant, Maximum strength, Rats

## Abstract

**Background:**

Creatine supplementation is known to exert an effect by increasing strength in high intensity and short duration exercises. There is a hypothesis which suggests that creatine supplementation may provide antioxidant activity by scavenging Reactive Oxygen Species. However, the antioxidant effect of creatine supplementation associated with resistance training has not yet been described in the literature. Therefore, we investigated the effect of creatine monohydrate supplementation associated with resistance training over maximum strength gain and oxidative stress in rats.

**Methods:**

Forty male Wistar rats (250-300 g, 90 days old) were randomly allocated into 4 groups: Sedentary (SED, n = 10), Sedentary + Creatine (SED-Cr, n = 10), Resistance Training (RT, n = 10) and Resistance Training + Creatine (RT-Cr, n = 10). Trained animals were submitted to the RT protocol (4 series of 10–12 repetitions, 90 second interval, 4 times per week, 65% to 75% of 1MR, for 8 weeks).

**Results:**

In this study, greater strength gain was observed in the SED-Cr, RT and RT-Cr groups compared to the SED group (*P* < 0.001). The RT-Cr group showed a higher maximum strength gain when compared to other groups (*P* < 0.001). Creatine supplementation associated with resistance training was able to reduce lipoperoxidation in the plasma (*P* < 0.05), the heart (*P* < 0.05), the liver (*P* < 0.05) and the gastrocnemius (*P* < 0.05) when compared to control groups. However, the supplementation had no influence on catalase activity (CAT) in the analyzed organs. Only in the heart was the CAT activity higher in the RT-Cr group (*P* < 0.05). The activity of superoxide dismutase (SOD) was lower in all of the analyzed organs in the SED-Cr group (*P* < 0.05), while SOD activity was lower in the trained group and sedentary supplemented group (*P* < 0.05).

**Conclusions:**

Creatine was shown to be an effective non-enzymatic antioxidant with supplementation alone and also when it was associated with resistance training in rats.

## Background

Creatine supplementation has been extensively studied since the 1990s and several studies [1-3] have analyzed its effects on maximum strength and body mass increase, which are well understood. The muscular storages of free creatine (Cr) and phosphorylated creatine (PCr) can be increased with creatine supplementation, leading to improvements in energy production by anaerobic systems in the first instances of physical exercises. Furthermore, this PCr increase can contribute to elevated protein synthesis and proteolysis prevention in muscular tissue [4].

From a different perspective, other studies have in investigated the antioxidant effect of creatine supplementation. In a cell-free experiment, the ability of creatine to quench reactive oxygen and nitrogen species, such as H_2_O_2_ and ONOO^−^, in muscle homogenates was observed [5]. On the other hand, the first study reporting antioxidant activity related to creatine supplementation in living cells was performed by Sestili and colleagues in 2006 [6]. However, few studies have assessed the antioxidant effect of creatine supplementation in biological systems, such as in humans or animals. A recent study pointed out the pleiotropic effects of creatine and its possible direct antioxidant effect in scavenging Reactive Oxygen Species (ROS) and Reactive Nitrogen Species (RNS) [7].

Oxidative stress and the subsequent damage to lipids, proteins and nucleic acids in acute response to aerobic exercise is well established in the literature [8-10]. In the same way, some studies have demonstrated an oxidative response when resistance exercises are performed [11-13]. Since systematic training can lead to increases in the activity of antioxidant enzymes (modulated by exercise adaptations) [14], it is still not clear whether Resistance Training (RT) can attenuate the acute oxidative damage experienced after exercise. Moreover, until now, there have been few studies that have evaluated the effect of creatine supplementation on resistance training maximum strength gain and oxidative stress.

Considering this, it is not clear whether creatine supplementation exerts intra and/or extracellular antioxidant effects and it plays a synergistic role in the adaptation of antioxidant enzymes associated with RT. Thus, the aim of this study was to evaluate the effects of monohydrate creatine supplementation associated, or not, with RT on oxidative stress and antioxidant enzymatic activity in the plasma, the heart, the liver and the gastrocnemius of rats.

## Materials and methods

### Animals

Forty male Wistar rats (250 to 300 g; 90 days old) from the UFCSPA Breeding Unit were divided into four groups: Sedentary (SED, n = 10), Sedentary + Creatine (SED-Cr, n = 10), Resistance Training (RT, n = 10) and Resistance Training + Creatine (RT-Cr, n = 10). The animals were housed under standard conditions (food and water *ad libitum*, temperature between 22 and 24°C, light–dark cycle of 12 hours).

The handling of the animals obeyed Law nº 11,794 of 10/08/2008, Law nº 6,899 of 07/15/2009, and Resolution nº 879 of 02/15/2008 (CFMV), as well as other provisions applicable to the use of animals for teaching and research, in particular the resolutions of the National Council on Animal Experimentation. This study was approved by CEUA/UFCSPA, under the protocol number 060/11.

### Resistance training protocol

Before the beginning of the experiment, the animals from the training groups were submitted to a familiarization period in the adapted squat apparatus designed by Krisan et al. [15]. The animals were placed in the apparatus and performed between 5 and 10 repetitions with 40% to 60% of their body weight, three times per week for one week. This load was considered low intensity as it has already been demonstrated that non-trained rats can lift up to three times their body weight upon first contact with the referred apparatus [16].

The rats were placed in a neoprene vest leaving them in bipedal position of the lower limbs. An electrical stimulus (4–5 mA, 0.3 seconds long, with a 3 second interval between each repetition) was applied in the rat’s tail using a surface electrode, in order to provoke the extension movement of the lower limbs of the rat, thus raising the load imposed in the squat apparatus. As this stimulus is considered low intensity, it is not expected to cause any physical injury to the animals [17]. All training sessions were performed in a dark room.

To determine the maximum lifted load in one repetition, the One Maximum Repetition (1MR) was utilized. From the obtained value, the load percentages required to perform the training protocol were determined. In response to training, strength gains were reported, making the realization of retests every two weeks necessary, in order to adjust the training load.

The training protocol lasted for a total eight weeks, at a frequency of four times per week. Each training session consisted of four series of 10–12 repetitions with a load of 65-75% of 1MR with a 90 second interval between each series [18]. The training program followed the guidelines of the American Physiological Society (2006) [19].

### Creatine supplementation protocol

The groups that were administered creatine monohydrate (presentation form: powder, purity: 99.9%, Delaware Laboratory, RS, Brazil) were given this by gavage solutions, as this resembles human oral consumption and ensures that the desired dose is achieved. The dosage of supplement administered followed the recommendations of the International Society of Sports Nutrition (2007) [20]. During the saturation period, which was the first seven days prior to the initiation of training, the dosage of 0.3 g/kg/day of creatine, diluted with 1.5 ml distilled water, was established. In the maintenance period, which comprised the last seven weeks, the dosage was set at 0.05 g/kg/day of creatine, which was diluted with 1.5 ml of distilled water. The animals received the supplement every day before training for the entire period of the protocol (including the days on which they did not train).

### Blood and tissues collection

The blood collection was performed through the decapitation method. The blood was stored in 2 ml Eppendorf microtubes containing EDTA and subsequently centrifuged (3,000 rpm for 10 minutes at 4°C) to separate the supernatant plasma. After blood collection, the collection of tissues (heart, liver and gastrocnemius) was performed, and samples were frozen at -80°C.

### Oxidative stress evaluation

#### Tissues homogenization

The heart, liver and gastrocnemius were defrosted, weighed in an analytical balance and homogenized in KPi buffer (KCl 1.15%), pH 7.4, with proportion of 9 ml/1 g of tissue. The proteases were inactivated with the addition of 0.5 mM phenylmethanesulfonyl fluoride (Sigma-Aldrich®, SP, Brazil) in anhydrous ethanol (1 μl of PMSF/1 ml of KPi buffer). The homogenization was performed manually in a glass macerator, with a Teflon pistil, counting 30 rotation movements and structure compression [21]. The homogenized samples were then centrifuged (3,000 rpm for 10 minutes at 6°C) and the supernatants utilized to determine the malondialdehyde (MDA), catalase (CAT) and superoxide dismutase (SOD) activities.

### Determination of total protein by the Bradford method

This technique is based in the interaction between the *coomassie brilliant blue* pigment BG 250 (Sigma-Aldrich®, SP, Brazil) and the protein macromolecules that contain aromatic or basic lateral amino acids. The interaction between the high molecular weight protein and the pigment provokes a shift of this in the equilibrium to the anionic form, which absorbs strongly at 595 nm [22]. To assess the dosage of protein in the tissue, 10 μl of homogenized sample was diluted in 190 μl of distilled water. Twenty microliters of this solution was placed in plastic cuvettes (optical path: 10 mm), containing 1 ml of Bradford reagent. The sample absorbances were determined at 595 nm, in a Lambda 35 spectrophotometer (Perkin-Elmer of Brazil, SP, Brazil). The protein standard curve was obtained from known concentrations of standard solutions of bovine albumin (1 mg/ml).

### Determination of malondialdehyde (MDA) through the thiobarbituric acid reactive substances test

To determine the MDA concentration, the technique according to JA Buege and SD Aust [23]. To promote the precipitation of proteins, 125 μl of tissue homogenate or plasmatic supernatant was added to 375 μl of 10% trichloroacetic acid solution. Next, the samples were centrifuged (3,000 rpm for 10 minutes at 6°C) and 250 μl of 0.670% thiobarbituric acid was added to 250 μl of supernatant. The solution was agitated and heated at 100°C in a water-bath for 15 minutes. After cooling, 750 μl of n-butanol was added. Then, following the second agitation, the samples were centrifuged (3,000 rpm for 5 minutes at 6°C). The stained supernatant was placed in glass microcuvettes to determine the absorbance at 535 nm in a Lambda 35 spectrophotometer (Perkin-Elmer of Brazil, SP, Brazil). The MDA concentration in each cuvette was expressed in nmol per mg of total proteins. To calculate the MDA concentration, the standard curve generated from the known concentrations of 1, 1, 3, 3-Tetrametoxypropane 100 nmol/ml in 1% H_2_SO_4_ solution was utilized.

### Determination of superoxide dismutase activity (SOD)

SOD activity was determined according to the technique of [24] at 420 nm. This reaction consisted of the inhibition of pyrogallol auto-oxidation by SOD activity. One unit of SOD is defined as the enzyme quantity capable of inhibiting 50% of the reaction. A total of 930 μl of TRIS buffer (TRIS 50 mM/EDTA 1 mM; pH 8.2), 4 μl of 30 μM catalase and 50 μl of homogenized tissue or plasmatic supernatant was placed into cuvettes. Then, 16 μl of 24 mM pyrogallol in 10 mM HCl was added to the solution. The sample absorbances were determined in a Lambda 35 spectrophotometer (Perkin-Elmer of Brazil, SP, Brazil), at 420 nm after 60 and 120 s. The results were expressed in SOD units/mg of total protein.

### Determination of catalase activity (CAT)

The CAT activity was determined through the decomposition of hydrogen peroxide at 25°C. In a quartz cuvette, 2865 μl of phosphate buffer 50 mM (pH 7.0) and 30 μl of homogenized tissue or plasmatic supernatant were added. Then, 35 μl of 0.02 M hydrogen peroxide was added to the solution. The sample absorbances were determined in a Lambda 35 spectrophotometer (Perkin-Elmer of Brazil, SP, Brazil), at 240 nm, and the results are expressed in pmol/mg of total protein [25].

### Statistical analysis

The data were evaluated using the software SigmaPlot version 12.0 for *Windows*. To detect a minimal difference of 18.91%, with an alpha error of 5% and a power of 80%, the minimal number of animals calculated to be required for each group was ten. This difference was based on a previous study in our laboratory, which utilized an outcome of maximum strength gain (Alves JP, personal communication, 2011). The results were expressed as the mean ± SD. Here, the two-way ANOVA test followed by the Student-Newman-Keuls’ *Post Hoc* test was used to make comparisons among groups. For associations among variables, the Pearson Correlation Test was performed. The accepted significance level was 5% (P < 0.05). For sample size calculations, the software SigmaPlot version 12.0 for *Windows* was utilized*.* To perform correlations and graphics, the software GraphPad 5.0 for *Windows* was used.

## Results

The body weight of the animals at the beginning of the study was similar (*P* > 0.05), but was different by the end. The trained groups demonstrated lower body weight gain when compared to the SED-Cr group (*P* < 0.01), while the RT group presented lower body weight gain compared to the SED and RT-Cr groups (*P* < 0.05).

### Maximum strength gain

**Figure 1:**
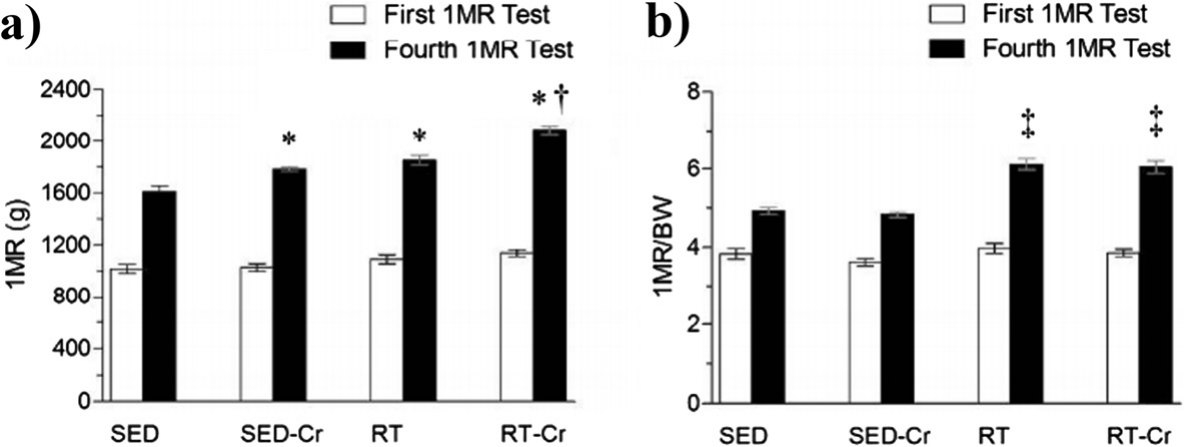
**Maximum strength gain after 8 weeks of intervention. a)** Absolute maximum strength gain related to the first to fourth tests of One Maximum Repetition (1MR); **b)** Relative maximum strength gain related to the first to fourth tests of One Maximum Repetition (1MR). Values in mean ± SD; n = 10 for all groups. SED, sedentary rats; SED-Cr, sedentary supplemented with creatine rats; RT, resistance training rats; RT-Cr, resistance training supplemented with creatine rats. Two way ANOVA, followed by the *post hoc* test of Student Newman-Keuls. *P < 0.001 vs. SED; †*P* < 0.001 vs. SED-Cr, RT; ‡*P* < 0.05 vs. SED, SED-Cr.

In relation to absolute maximal strength gain (Figure 1a), a higher strength gain was observed in the creatine supplemented groups and in the group only submitted to RT, compared to the SED group (*P* < 0.001). The RT-Cr group presented higher maximum strength gain when compared to other groups (*P* < 0.001).

When the analysis related to body weight and maximal strength gain was performed (Figure 1b), a higher strength gain was only observed in the trained groups when compared to the sedentary groups (*P* < 0.001).

### Oxidative stress and antioxidant enzymes activity

**Figure 2:**
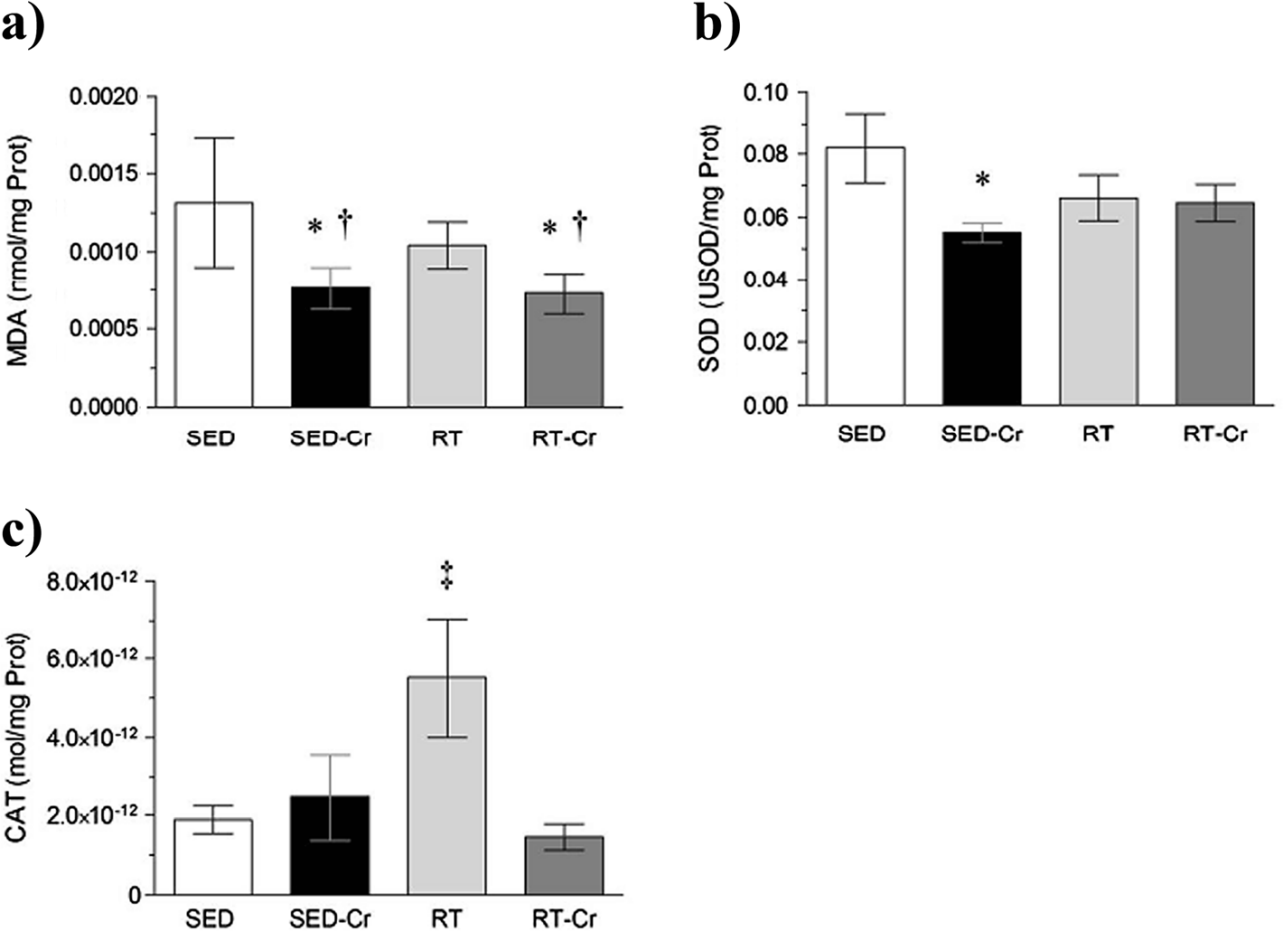
**Oxidative stress in plasma after 8 weeks of intervention.** Concentrations of **a)** MDA in plasma; **b)** SOD activity in plasma; and **c)** CAT activity in plasma. Values in mean ± SD; n = 10 for all groups. SED, sedentary rats; SED-Cr, sedentary supplemented with creatine rats; RT, resistance training rats; RT-Cr, resistance training supplemented with creatine rats. Two way ANOVA, followed by the *post hoc* test of Student Newman-Keuls. *P < 0.05 vs. SED; †*P* < 0.05 vs. RT; ‡*P* < 0.05 vs. all groups.

With regard to the plasma concentration of MDA (Figure 2a), a lower concentration was observed in the creatine supplemented groups, when compared to the SED and RT groups (*P* < 0.01). The activity of plasmatic SOD (Figure 2b) was lower in the SED-Cr group, compared to the SED group (*P* < 0.05), but there were no differences between trained groups. The activity of plasmatic CAT (Figure 2c) was only higher in the RT group in relation to other groups (P < 0.05). No correlation was observed between SOD activity and MDA concentration in plasma (r = 0.0321; *P* > 0.05).

**Figure 3:**
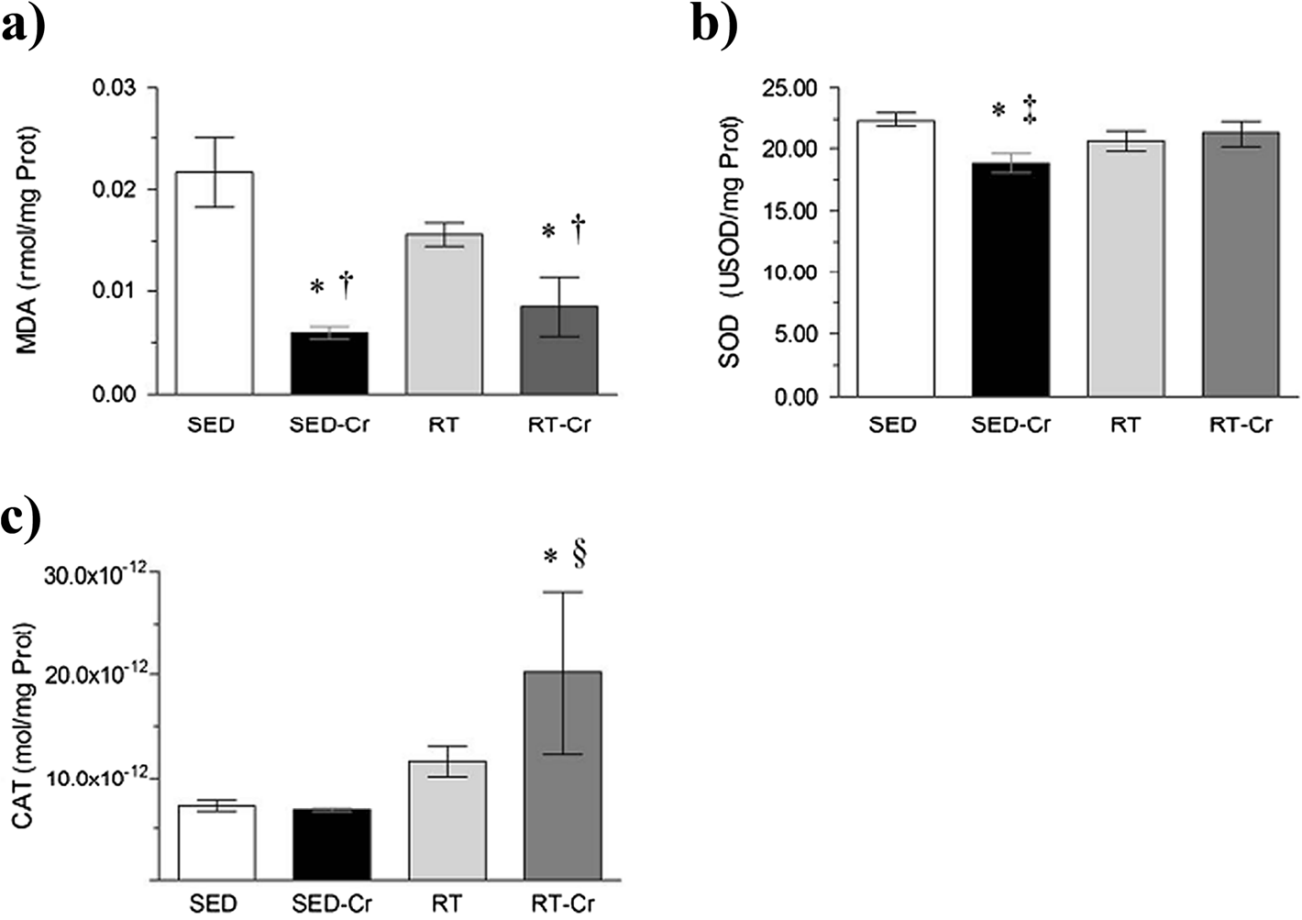
**Oxidative stress in heart after 8 weeks of intervention.** Concentrations of **a)** MDA in heart; **b)** SOD activity in heart; and **c)** CAT activity in heart. Values are mean ± SD; n = 10 for all groups. SED, sedentary rats; SED-Cr, sedentary supplemented with creatine rats; RT, resistance training rats; RT-Cr, resistance training supplemented with creatine rats. Two way ANOVA, followed by the *post hoc* test of Student Newman-Keuls. *P < 0.05 vs. SED; †*P* < 0.05 vs. RT; ‡*P* < 0.05 vs. RT-Cr; §*P* < 0.05 vs. SED-Cr.

Likewise, in relation to the heart concentration of MDA (Figure 3a), a lower concentration was observed in the creatine supplemented groups compared to the SED and RT groups (*P* < 0.01). The activity of SOD in the heart (Figure 3b) was lower in the SED-Cr group compared to the SED and RT-Cr groups (*P* < 0.05), but there were no differences seen with the RT group. The CAT activity in the heart (Figure 3c) was only higher in the RT-Cr group, in relation to sedentary groups (*P* < 0.05). Also, a positive correlation was observed between SOD activity with MDA concentration in the heart (r = 0.4172; *P* < 0.05).

**Figure 4:**
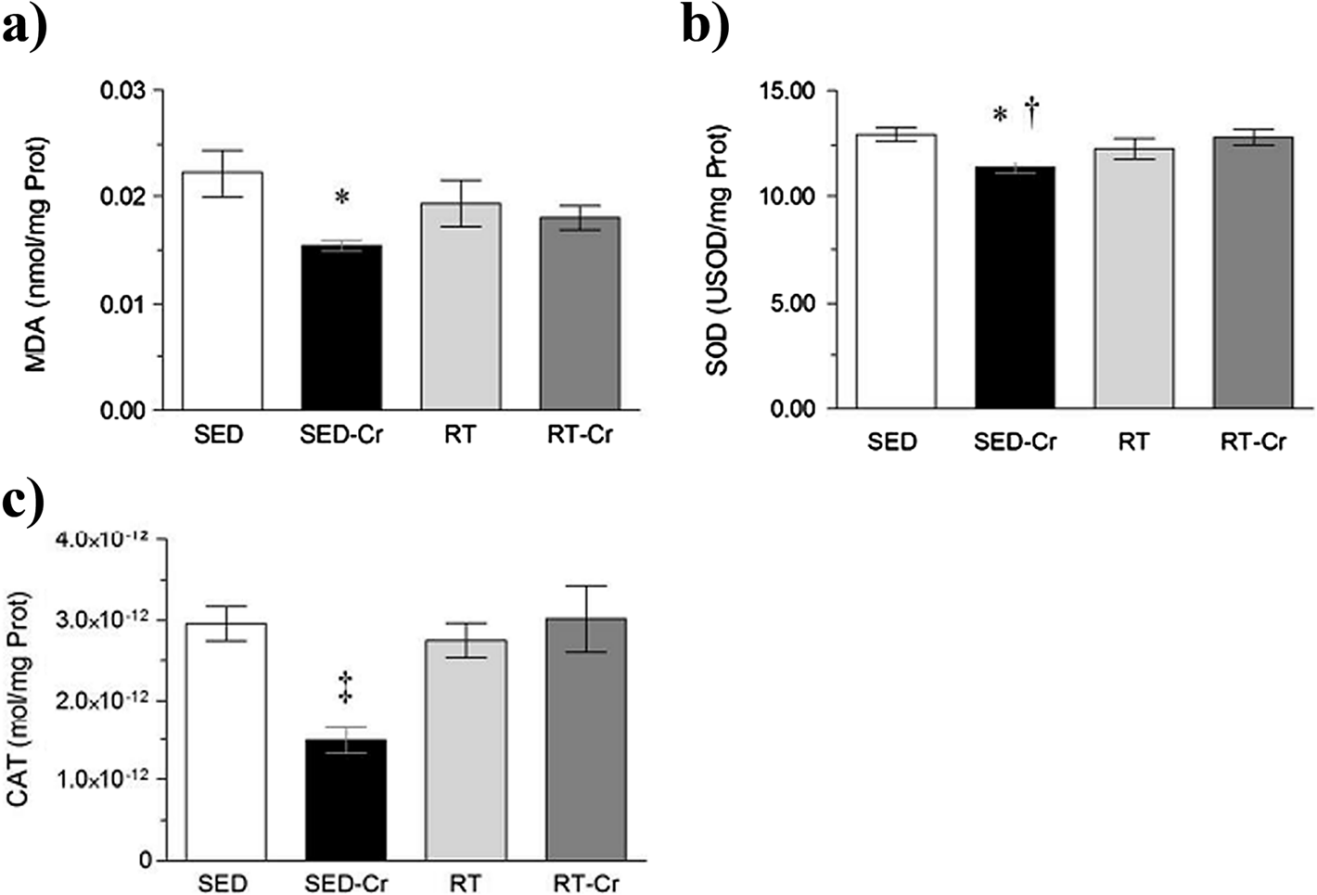
**Oxidative stress in liver after 8 weeks of intervention.** Concentrations of **a)** MDA in liver; **b)** SOD activity in liver; and **c)** CAT activity in liver. Values in mean ± SD; n = 10 for all groups. SED, sedentary rats; SED-Cr, sedentary supplemented with creatine rats; RT, resistance training rats; RT-Cr, resistance training supplemented with creatine rats. Two way ANOVA, followed by the *post hoc* test of Student Newman-Keuls. *P < 0.05 vs. SED; †*P* < 0.05 vs. RT-Cr; ‡*P* < 0.001 vs. all groups.

In the liver, only the SED-Cr group demonstrated a lower MDA concentration (Figure 4a) in relation to the SED group (*P* < 0.05), without any differences reported between the trained groups. The SOD activity in the liver (Figure 4b) was lower in the SED-Cr group when compared to the SED and RT-Cr groups (*P* < 0.01). CAT activity in the liver (Figure 4c) was higher in the SED and trained groups in comparison with SED-Cr group (*P* < 0.01). A positive correlation was observed between SOD activity and MDA concentration in the liver (r = 0.3722; *P* < 0.05).

**Figure 5:**
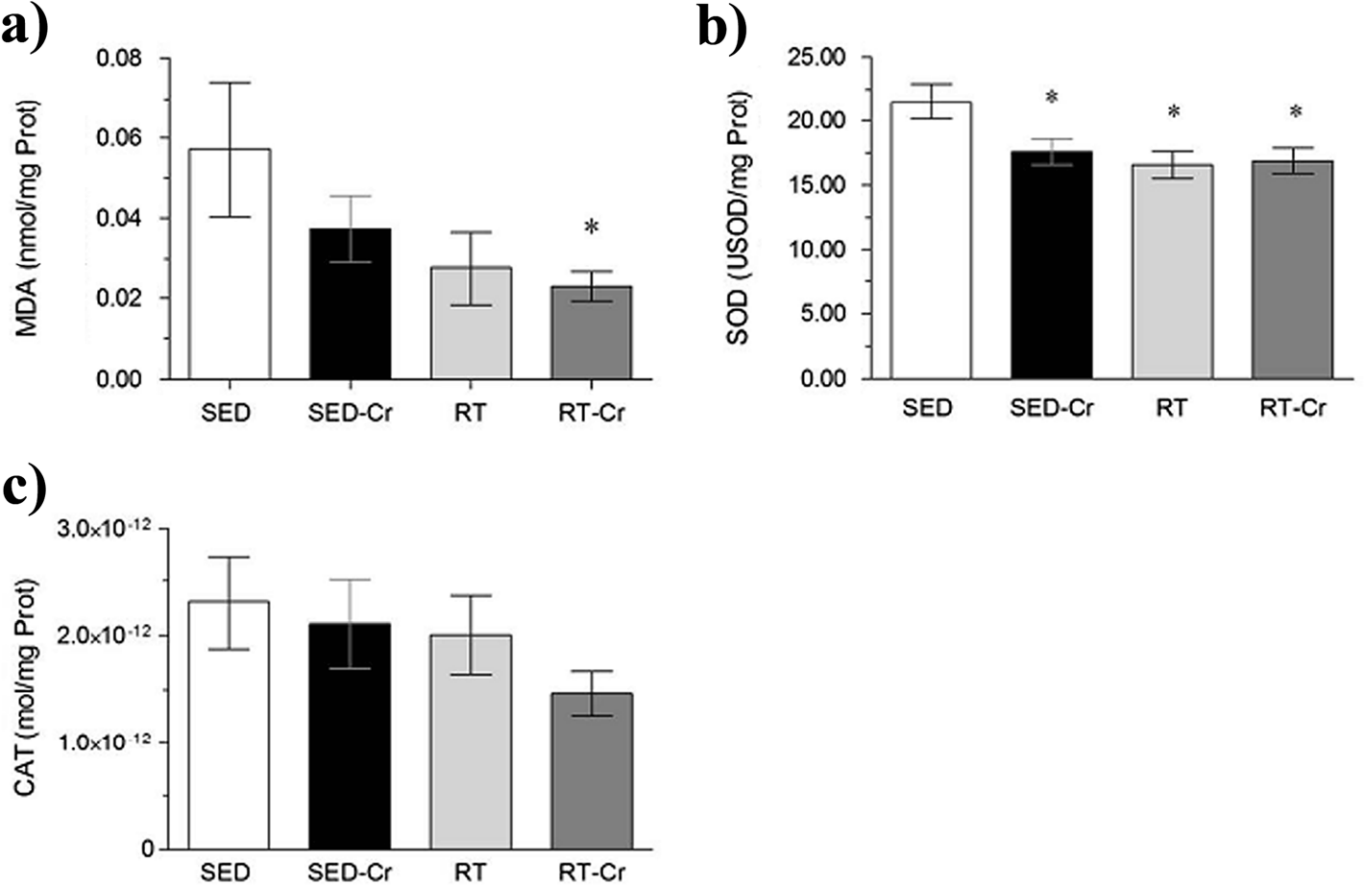
**Oxidative stress in gastrocnemius after 8 weeks of intervention.** Concentrations of **a)** MDA in gastrocnemius; **b)** SOD activity in gastrocnemius; and **c)** CAT activity in gastrocnemius. Values in mean ± SD; n = 10 for all groups. SED, sedentary rats; SED-Cr, sedentary supplemented with creatine rats; RT, resistance training rats; RT-Cr, resistance training supplemented with creatine rats. Two way ANOVA, followed by the *post hoc* test of Student Newman-Keuls. *P < 0.05 vs. SED.

Considering the MDA concentration in the gastrocnemius (Figure 5a), only the RT-Cr group presented a lower concentration when compared to the SED group (*P* < 0.05). SOD activity in the gastrocnemius (Figure 5b) was lower in the trained and SED-Cr groups compared to SED group (*P* < 0.01). No differences were observed among groups in relation to CAT activity in the gastrocnemius (*P* > 0.05) (Figure 5c). Also, no correlation was observed between SOD activity and MDA concentration in the gastrocnemius (r = 0.0283; *P* > 0.05).

## Discussion

This is one of the first studies to demonstrate a possible antioxidant effect of creatine supplementation either in association or not with an RT protocol. It is also one of the few studies to elucidate the antioxidant effect paradigm of creatine *in vivo*.

In our study, after 8 weeks of RT in squat apparatus adapted for rats, a significant increase in the maximum strength was observed in all groups. However, the strength was higher in the trained group supplemented with creatine. Similar results were observed in other studies that evaluated the gain of maximum strength in humans [26-28]. Although it has not been evaluated in the present study, the muscular content of free creatine and creatine-phosphate storage appear to contribute to an increase in the maximum strength of creatine supplemented individuals submitted to the RT protocol, as demonstrated by Buford and colleagues in 2007 [20].

In the present work, a lower plasmatic lipoperoxidation, evaluated by MDA, was only observed in those groups which received creatine supplementation. These results are in contrast to those found by Kingsley and colleagues [29], who sought to evaluate serum lipid peroxidation in humans submitted to two tests until exhaustion using a cycloergometer and did not observe any influence from creatine supplementation on this marker of oxidative stress. In the previous study, the volunteers were physically active and only familiarized to an exhaustive exercise protocol. However, in our work, we submitted the animals to a resistance training program which led to different muscular and metabolic adaptations. Deminice and colleagues [30] evaluated the acute effect of creatine supplementation for 7 days on plasma oxidative stress in humans submitted to sprint exercise; no antioxidant effect was observed. The divergence from the results presented here might be explained by the different types of exercise, such as the hemodynamic response and the predominant energetic metabolism related to resistance exercise compared to that reported for sprinting or cycling. In another study, rats submitted to 1-h of swimming (load of 4% of body weight) and supplemented with creatine (2% of diet) for a period of 28 days, showed a reduction in plasmatic TBARS immediately after exercise, and 2 h and 6 h after the swimming exercise [31]. It is possible that a longer loading phase of creatine supplementation can increase the antioxidant status, rather than a shorter period of loading. However, when it is associated with a training regimen, higher effects were observed for plasma lipoperoxidation [32]. Interestingly, similar results were observed in the present study. In this way, this antioxidant effect of creatine supplementation associated with RT in plasma oxidative stress corroborated our findings.

Since the SED-Cr group presented a reduction in plasma activity of SOD enzyme and lower lipoperoxidation, it is possible that the creatine may have acted as an ROS scavenger. In the same way, supplemented groups showed no increase in CAT activity; this only occurred in the group submitted to RT. CAT is an enzyme that is highly modulated by physical training, especially by endurance training, where the formation of ROS by the leakage of superoxide radicals in the electron transporter chain is much higher due to the greater utilization of the oxidative pathway [33-35]. Since, in our results, plasmatic CAT activity was higher in the RT group, it is possible that it is necessary to increase this antioxidant enzyme (due to the lack of non-enzymatic antioxidants like creatine) in order to reduce the plasma lipoperoxidation in this group.

Creatine has been considered a cytoplasmic antioxidant of direct action that would mainly promote the scavenging of ROS superoxide radicals [36]. Recently, Lygate and colleagues [37] sought to assess a possible protective effect of creatine in the ischemia-reperfusion process in mice submitted to acute myocardial infarction. The cardiomyocytes were exposed to an oxidant agent, H_2_O_2_, in order to evaluate the antioxidant action in the fluorescent pigment. Creatine treatment was not able to attenuate the damage promoted by H_2_O_2_.

However, in our work, a reduction of lipoperoxidation was demonstrated in the heart tissue of creatine supplemented groups, suggesting a possible direct antioxidant effect in the suppression of superoxide radicals in the heart, probably due to its chemical affinity as an antioxidant, being higher with this specific ROS [5,6].

An interesting finding of our study was that, in the heart, SOD activity was reduced in the sedentary group that was supplemented with creatine, in comparison to both the control group and the RT creatine supplemented group. This was in accordance with Siu and colleagues [38], where low intensity exercise (walking) for 8 and 20 weeks was not able to increase SOD activity in the heart of rats. Resistance exercise is characterized by a pressure overload in the heart during its execution, causing an increase in cardiac muscle mass [39]. This suggests that, in part, the RT-Cr group increased SOD activity as an adaptive response to a higher formation of anion superoxide in this tissue under physical training conditions, and that the increased production of this ROS occurs through the xanthine oxidase pathway [40,41]. Creatine supplementation may have exerted a synergistic effect with RT in relation to SOD activity modulation in the heart. In chronic-progressive stress conditions, and in RT, supplementation appears to exert a synergistic effect with regard to adaptation to RT with creatine supplementation, involving the cellular signaling enzymatic adaptation of SOD in cardiac tissue. This mechanism occurs via activation of the NAD(P)H oxidase system that, through vasoactive (angiotensin II) and inflammatory mediators (IL-6, TNF-α), modulates the expression of antioxidant enzymes in a short period [42,43].

CAT activity in cardiac tissue seems to be modulated by the interaction of creatine supplementation with RT, as observed by McClung and colleagues [44], who evaluated the effect of the association of creatine with high intensity exercise on cardiac function in rats and found that this interaction was able to up-regulate the cardiac functional capacity. These results indicate a possible direct or indirect enzymatic modulation of creatine in synergism with training.

As creatine is not synthesized exclusively in the kidney and in the pancreas, but at higher proportions in the liver, and is then mainly transported to the skeletal muscle, we investigated the liver with the aim of developing a hypothesis about the redox state of this organ in the presence of supplementation, either associated or not with resistance training. Our results are different to those found by Radak and colleagues [45], who reported an attenuation of lipoperoxidation levels in the animals submitted to treadmill running training which was adapted for rats. The difference in training protocols, age and animal species may have directly influenced the difference between the results obtained and those of our study.

Studies that have evaluated the effect of creatine supplementation on oxidative stress in different structures are very limited. In our study, we found lower SOD activity in both heart and liver. Botezelli and colleagues [32] evaluated lipid peroxidation, SOD and CAT activity in the liver following three different training protocols (aerobic, strength and concurrent). However, the training did not have any influence on antioxidant enzymatic activity. Creatine seems to have the same response in different tissues, since the increased production of ROS and RNS at the expense of strength exercise possibly acted upon cellular signaling to increase antioxidant enzymatic defenses [46].

When we analyzed the lipoperoxidation in skeletal muscle, we observed that only the RT-Cr group showed lower oxidative damage compared to the SED group. Similar results were found by Guimaraes-Ferreira and colleagues [36], since creatine supplementation associated or not with RT did not change the CAT and SOD activity in skeletal muscle. In this tissue, creatine seems to exert a scavenging antioxidant effect and does not act as an antioxidant enzymatic activity modulator. In a model of spontaneously hypertensive rats submitted to a creatine supplementation protocol, it has been demonstrated that this supplementation does not promote the attenuation of oxidative stress in skeletal muscle [47].

Lastly, this was one of the first studies to evaluate the effects of isolated creatine supplementation or that associated with RT on oxidative stress. As a limitation of this work, it can be noted that a few antioxidant enzymes (e.g. glutathione peroxidase, glutathione reductase, peroxiredoxin), non-enzymatic antioxidants (e.g. glutathione, GSH/GSSG ratio, total antioxidant capacity), biomarkers of oxidative damage (protein carbonyl, 8-OH-dG) and/or activity of ROS and RNS were not analyzed, but this could clarify certain results obtained in the present study.

## Conclusions

The supplementation of creatine monohydrate along with 8-week RT was able to reduce oxidative stress. In addition, SOD activity was positively influenced by creatine supplementation in all of the organs analyzed. The supplementation did not influence CAT activity in all organs similarly, except for in the heart. However, further *in vivo* studies associating creatine supplementation with RT are necessary to confirm the findings of this study.

## Competing interests

The authors declare that they have no competing interests.

## Authors’ contributions

SGP, NRB, DZA, AJP conception and design of research; SGP, NRB, DZA, AJP, POM, DDM performed experiments; SGP, NRB, DZA, AJP, POM, DDM, RRC, LPD analyzed data; SGP, NRB, DZA, AJP, POM, DDM, RRC, LPD interpreted results of experiments; SGP, NRB, DZA, AJP prepared figures; SGP, NRB, DZA, AJP, POM, DDM, RRC, LPD drafted manuscript; SGP, NRB, DZA, AJP, POM, DDM, RRC, LPD edited and revised manuscript; SGP, NRB, DZA, AJP, POM, DDM, RRC, LPD approved final version of manuscript. All authors read and approved the final manuscript.
